# Selected Medical Cases at the Buffalo Hospital of the Sisters of Charity

**Published:** 1849-10

**Authors:** Austin Flint

**Affiliations:** Attending Physician


ART. II.—*Selected Medical Cases at the Buffalo Hospital of the Sisters of Charity.* By AUSTIN FLINT, M. D., Attending Physician.

CASE 1. *Masked Intermittent; cerebral oppression, approaching coma; absence of chills and appreciable febrile movement.*

George Brown, mariner, aged 22, unmarried, entered Hospital Oct. 5, P. M., 1848.

States that he has been ill three weeks. Was attacked with intermittent fever of the tertian type. Kept about for some time, but does not recollect how long it was before he took to his berth. He appears unable to give an accurate account of his history; seems to understand the import of questions, but loses himself before he finishes his replies. Answers, at times, incoherently.

*Present Symptoms:* Tongue moist and furred; abdomen not distended, no tenderness on pressure, and no eruption; skin cool; pulse 80.

Treatment:—S. Morph. gr. 1-8,
P. Camph. grs. iv.
Every six hours.

After convalescence was established, the following account of his previous history was furnished by the patient:—He was ill three weeks before entering Hospital. He was attacked on board a schooner, in the Portage river, Ohio, sixteen miles from the Lake, with Tertian Intermittent. He kept about the vessel for the first week; was unable to work much, but did a little. At expiration of the first week consulted a Physician on shore, who gave him a powder, and after taking it, he vomited frequently, and severely, during the entire day. He also gave him six powders of Quinia, which he took after the vomiting, one every six hours. After this he had no chills, but was confined to his berth. Retains a very imperfect recollection of what transpired during the fortnight that elapsed between his taking to his berth, and his arrival at Buffalo, when he was at once transferred to Hospital. During that fortnight he was very feeble; had no appetite, taking nothing but tea and a piece of orange, except on one occasion a custard. Had profuse diarrhœa part of the time; one day he was actively delirious. Does not remember vomiting but once after taking the medicine. Took no medicine during the fortnight referred to. Recollects when the vessel arrived at Buffalo. Rode from the Dock to Hospital, (distant a mile and a quarter,) in a cab, and had full consciousness during the ride, but of what occurred directly after reaching Hospital retains no recollection. Recollects nothing that occurred for the first five days after entering Hospital. The first thing that he recollects was his taking beef essence, which he disliked.

Oct. 6.—This patient last evening was restless and entirely unconscious. Rolled out of bed twice. Did not reply to questions, and appeared not to understand their import. He continued somnolent most of the night. The pulse during the night was of natural frequency, and skin warm. This morning answers questions readily and coherently. He has a vacant look. He appears greatly prostrated. Tongue moist and coated. Pulse 76; skin of natural temperature; no dejection. Abdomen not distended; no tenderness on pressure; no eruption.

Treatment:—S. Quiniæ, grs. ij.
S. Morph. gr. 1-8.
Pulv. Camph. gr. iij.
Every four hours.

Essence of beef, and thin milk porridge, for diet.

P. M.—This patient now lies in nearly an unconscious state, with eyes partially closed; opens his eyes on being addressed, but returns no answers to questions, and manifests no comprehension of their import. He cannot be made to protrude his tongue. Flies creep over his face without disturbing him. Slight stertor in respiration. Temperature of skin moderately elevated. Pulse 84, well developed. No sordes; no dejection. Been in this state most of the day.

Treatment:—Morphia discontinued, quinia and camphor continued, every two hours. Nutriment and stimulus to be given at short intervals.

Oct. 7, A. M.—The symptoms detailed in the foregoing record continued until about 8 ½ o'clock, P. M., when, after having taken about two table spoonfuls of brandy, with nutriment, he appeared to revive; seemed conscious of what was going on about him, and asked for drink. The brandy was continued through the night. This morning he replies to questions, and protrudes his tongue. Says he feels pretty well, and would like some toast for breakfast. Urinates with difficulty, discharging a little at a time. No dejection since his admission. Seems indifferent to persons and things around him. Flies continue to creep over his face without disturbing him. Pulse 80, well developed; skin warm, and somewhat moist. Perspired during the night.

Treatment:—Ol. Ricini ℥ss, and continue quinia and camphor.

P. M.—Lies with his eyes open, but pays no attention to any thing said to him, and does not turn his eyes when addressed. Skin cool and moist. Pulse 88. Respiration tranquil. The symptoms assumed their present character at about 2 o'clock, P. M. Cont. med., and give brandy, ʒj, every hour, with nutriment.

Oct. 8, A. M.—He continued perfectly insensible until about 5, A. M. He began to sweat at 1, A. M., and sweat profusely. Bowels were evacuated unconsciously in bed. Considerable difficulty was experienced in giving food, drink, and medicine, during the night, owing to difficulty of deglutition. Passed urine in bed.

This morning he appears conscious, but greatly prostrated. Tongue thickly coated, skin moist and cool. Pulse 72.

Treatment:—The powders of quinia and camphor to be continued, hourly, until 2, P. M. Cont. brandy, and nutritious diet.

P. M., 8 o'clock.—Brown preserves as much consciousness as was manifested this morning. Protrudes his tongue, and replies to questions. Difficulty of deglutition continues, rendering it not easy to administer remedies and diet; skin cool and moist. Pulse not accelerated. Says he feels tired. No dejection, nor passage of urine to-day. He recognised a shipmate who called to see him this afternoon.

Treatment:—Continue quinia and camphor every two hours. Brandy and diet as before.

Oct. 9, A. M.—Appears to be rational this morning. Answers questions more promptly than hitherto; was wakeful during the night, and rational. Tongue coated and moist; skin normal. Pulse 60. Urinated very copiously in bed this morning. Desires toast. No dejection.

Treatment :—Cont. med. every two hours, until 2, P. M.

Oct. 10, A. M.—Passed a comfortable night; aspect bright. Appears quite rational; tongue cleaning; skin normal. Pulse 52. Has eaten toast this morning with relish.

P. M.—Perfectly conscious. Has passed urine copiously.

Oct. 11, A. M.—Aspect bright. Tongue cleaning; skin warm. Pulse 48. One dejection. Ate toast for breakfast with great relish.

Treatment:—Cont. Quinia, omitting the camphor. Brandy every four hours.

Oct. 12.—Improving, Pulse 48. Ate toast, egg, and coffee for breakfast.

Treatment:—S. quinia, grs. ij, three times daily. Discontinue brandy. From this date he continued to convalesce, without any untoward symptoms, and was quite well, although somewhat weak, on the 28th of October. He left the Hospital on the 20th of November, perfectly well.

*Remarks:* This case certainly is novel and interesting. The patient was first attacked with *intermitting fever*, which, from want of proper care, was converted into a *remittent*. The latter pursued its career without medical treatment, and under unfavorable circumstances, the patient being on board a small vessel, without proper diet, and nursing, as well as remedies. From the history given by the patient, the remitting fever was of that character which may be distinguished as *typhoid remittent*, and which is sometimes mistaken for typhoid continued fever. The phenomena of the two forms of fever were probably blended. Finally, at the time of his entering the Hospital, the disease assumed (as it often does) an intermittent form. But, instead of regular paroxysms of chills, febrile movement, and perspiration, the occurrence of the paroxysms was characterised by the diurnal recurrence of entire insensibility, continuing for ten or twelve hours, and terminating by profuse perspiration. The diagnosis was rendered somewhat difficult, not only from the imperfectness of the history obtained prior to the patient's convalescence, but by the apparent non-excitation of the pulse, and absence of febrile movement, and also by the fact, that, in the apyrexial period, the mind was morbidly dull. The non-excitation of the pulse was subsequently, in a measure, accounted for, by the natural pulse being found to be below the average in frequency. A case of this description might readily enough fail to be diagnosticated, at the stage in which it came under observation, if the patient were not seen at different periods of the day, and the symptoms carefully watched. It is not too much to say, that without the treatment following an early diagnosis, the case must have soon terminated fatally. The condition of the patient was apparently so hopeless during the paroxysm, and so great was the difficulty of administering remedies and nutriment, that the attendants were disposed to abandon, on one occasion, farther efforts of relief, but, on being encouraged to persevere, their attentions were unremittingly continued, and, as the result proved, with most gratifying success. It is probable that the case would have terminated fatally under more unfavorable circumstances as regards care and nursing. As it was, but little expectations were entertained of his recovery. The administration of the specific remedy for periodic fevers was probably essential to the favorable issue of the disease. It may fairly be doubted if such would have been the issue, had the cerebral phenomena been attributed to pure congestion, or to an ordinary typhoid condition, and been treated accordingly. Depletory treatment, there is every reason to suppose, would have been most unfortunate. The immediate efficacy of the quinia, continued in doses of grs. ij, hourly, in the apyrexia, was striking.

A similar variety of masked intermittent has not been unnoticed, although it is extremely rare. Andral, in his *clinique medicale,* gives the history of a case in which coma periodically recurred, and was treated as a case of simple apoplexy, until the regularity of succession in the attacks, led to the suspicion of the true character of the affection, and the efficacy of the treatment adopted in conformity with this suspicion, confirmed its correctness.

As regards the pathology of cases of this description, I will only say, they are not to be placed in the category with those cases of pernicious intermittent, occasionally met with, in which true apoplectic coma complicates either the first or second stage of the disease. In the latter instance the cerebral oppression is incident to the paroxysm, and is probably due, chiefly, to disordered circulation within the cranium. In the former, the cerebral oppression takes the place of the ordinary phenomena of the paroxysms, and it may be reasonably doubted if it is attributable to mere congestion.

The only case resembling that which we have given, which had previously fallen under our notice, was a case in which, in a female, subject to intermittent fever, shortly after the time of the second recurrence of a paroxysm, there occurred a hysterical attack, occasioned by slight convulsions at first, and followed by insensibility of about twelve hours' duration. The patient, in this case, was taking the specific remedy for intermittent fever, and did not experience any recurrence of the disease after the hysterical attack.

Case II. *Common continued (Typhoid) Fever, Peritonitis. Death. Autopsy; Cicatrization of ulcerations of ileum.*

For the purposes of this report, it will suffice to give an account of the progress of the case in a few words.

Joseph Peng, German, aged 43, cooper, entered Hospital Dec. 19, 1848. He had arrived at New York, from Germany, on the 5th ult., and had been four weeks in Buffalo. Had been ill eleven days before entering Hospital. Was carried to the Poor house four days after becoming ill, whence he was transferred to Hospital. He presented the phenomena of continued fever. Was delirious during night for several days. Tongue dry, glazed, and fissured. Moderate diarrhœa. Pulse 88. Moderate tympanites, and considerable abdominal tenderness, on pressure, especially marked in the iliac region of each side. In the progress of the case the delirium disappeared, tenderness over abdomen diminished, and he gave indications of convalescence. The pulse, however, continued accelerated; some abdominal tenderness continued; diarrhœa continued, and was much increased; vomiting occurred frequently, singultus became a troublesome and obstinate symptom, and death, finally, occurred on the 4th of January, 1849, *seventeen* days after his admission, and *twenty-eight* days from the date of the commencement of the disease.

The post-mortem examination, from necessity, was limited to the abdomen, and even here was cursory. The omentum was adherent to the intestines, and the convolutions adherent to each other. These adhesions were generally easily broken up, but occasionally they were quite firm, and the force required for separation occasioned rupture of the intestinal tunics. In several instances, in separating the firm adhesions uniting the intestines together, pus, in small quantities escaped. On longitudinal section of the small intestine, ulcerated patches, completely cicatrized, were found situated near the ileo-coecal valve; one of these patches was as large as a quarter of a dollar. Sharp, and somewhat elevated edges of mucus membrane defined the boundaries of the cicatrized ulcers, and the latter were covered with a delicate expansion of cellular tissue. Along the ileum for the space of several feet, were numerous ulcerated patches, varying in size and form, which were mostly cicatrized as just described. In some, situated highest in the tube, the cicatrizing process was not entirely completed. All were situated in the elliptical plates formed by the agminated glands of Peyer. The mesenteric glands were enlarged, but none were observed larger than a large pea. On dividing several, no pus was discovered.

*Remarks:* The interest of this case consists in the illustration which it afforded of complete and progressive cicatrization of the intestinal ulcerations characterizing common continued, or Typhoid fever. That many cases of this disease, terminating favorably, must have been complicated with ulcerations which become entirely healed, is almost certain, as an inferential conclusion, but it is not often that we have an opportunity of examining the healing process in the stages in which it was presented in this case. The appearances were similar to those described by other observers. The ulcerated spaces are first covered by a delicate serous-looking membrane, which gradually becomes converted into mucus tissue, after which the fact of the previous existence of ulceration is less demonstrable.

The patient, in this case, died of chronic peritonitis complicating fever. The symptoms during life did not denote the existence of disease of the thoracic or cerebral organs.

CASE III. *Continued (Typhus) Fever complicated with Parotis.*

Edward Darling, aged 23, American, mariner, unmarried, entered Hospital March 29th, 1849.

On the 20th inst. he was exposed to cold and wet, and became chilled. Previously to this he was quite well. Directly afterward he had loss of appetite, and general *malaise,* which increased daily, until the 24th, when he took an emetic, and, from that date, was confined to the bed. From that time he has had fever. He is unable to give a definite account of his symptoms. Has been attended by Dr. John S. Trowbridge prior to his entering Hospital, who regarded the case as one of Typhus fever.

On his entrance, yesterday afternoon, he had febrile movement, and some confusion of ideas. I prescribed S. Morph. gr. ⅛, Spts. Nitre Dulc. ʒj, every four hours. This morning he presents considerable enlargement of the parotid gland, and investing tissues, of the right side, the lower portion of the ear being thrown outward, as in mumps. The swelling is quite tender and painful. He cannot, without pain and difficulty, open the mouth. Skin cool; pulse 100, thrilling; tongue moist; mind seems clear; no thirst; says he had some good sleep during night. Had several dejections during night.

Treatment:—S. Quiniæ, grs. ij. P. Doveri, grs. iij, every four hours. Apply to the affected parotid, warm Lin. Sapo cum Opio. If he suffers much from pain, give S. Morph. gr. 1-6, and repeat *pro re nata.*

March 31st.—Aspect bright. Retains muscular strength sufficiently to get out of bed unaided. No muscular tremor. Parotid greatly swelled and hard, but less tender and painful. Cannot open his mouth sufficiently to protrude his tongue. Replies to questions coherently. No indications of deafness. Abdomen and chest covered with eruption of a dark red color, not elevated, redness not disappearing on pressure. Eruption extends to upper and lower extremities. Abdomen not distended, nor tender. Dejection in night. Urine scanty and high-colored, without sediment. Skin cool. Pulse 120, small and feeble.

Treatment:—Cont. Quinia. Give brandy, ℥ss., every three hours. For diet, milk porridge, toast, and beef essence.

April 1.—Pulse 130, swelling of parotid the same. Evinced mental delusions during night. Declares he is better. No dejection; some cough and expectoration. Eruption fading.

P. M.—Pulse 136, other symptoms same. During absence of attendant he got out of bed, and succeeded in dressing himself, even to putting on his boots. Thought he would "try sitting up awhile." Does not mutter, and usually replies to questions coherently.

Treatment:—Poultice to Parotid. Tart. Ant. et Pot gr. $\frac{1}{8}$, S. Morphiæ gr. $\frac{1}{8}$, every two hours, to be omitted if vomiting or nausea occur. Omit brandy.

April 2.—No material alteration in symptoms, or treatment.

April 3d.—Reports "middling." Aspect bright. Disposed to somnolency, but easily roused. Skin warm; pulse 120. Abdomen not distended; no tenderness, nor gurgling. No sordes. Able to move himself readily in bed. Replies coherently, but, at times, evident mental aberration. Moderate cough and expectoration. Swelling of Parotid distinctly fluctuating below the ear. No dejection for several days.

Treatment:—Enema of ol. Ricini and warm water. Cont. poultice to Parotid. S. Quiniæ, gr. ij, every four hours. Omit antimony, and give a little brandy in lemonade. Cont. diet as heretofore.

P. M.—Skin hot. Pulse 130, strong, and vibratory. Moderate epistaxis. Somnolent most of the day. Opened abscess below the ear, free purulent discharge following.

Omit treatment directed this morning, and substitute Tart. Ant. et Pot. grs. iij, S. Morph. gr. ss., aquæ ℥ij; ʒj hourly, unless it nauseates. No dejection. Enema to be repeated.

April 4.—Passed a comfortable night. Skin and tongue moist. Free dejection after enema; and, subsequently, dejection in bed, involuntarily. Abdomen soft. Eruption nearly gone. Antimony produced no nausea. Abscess discharges freely. Pulse 64.

Treatment continued.

P. M.—Pulse 120. Abdomen moderately tympanitic. Some sordes Somnolent, but readily roused. Some subsultus. No dejection. Refused nourishment to-day, saying that he could not retain it, but has not otherwise complained of nausea.

Omit antimony. Emollient enema. Carb. ammoniæ grs. iij, every two hours. S. Quiniæ, grs. ij, every four hours.

April 5.—Aspect better. Skin moist and cool. Pulse 64, soft and regular. Respirations 24, equal. Two dejections; abdomen soft. Free discharge of urine, high-colored, without deposit.

P. M.—Pulse 76, rather quick and forcible. Large dejection, yellow. Abdomen flaccid. Disposed to somnolency, and sleeps rather heavily. Respirations 24.

Continue treatment.

April 6.—Exacerbation of fever last evening, accompanied by delirium. General aspect good. Pulse 94, soft, regular. Respirations 28, somewhat heavy, with some dilatation of alæ nasi. One dejection. Swelling of Parotid diminished; continues to discharge. Cough and expectoration continue.

Cont. Treat. Let him have chicken broth, and a wine glass of ale every four hours.

April 7, A. M.—Symptoms continue the same. At P. M. somnolent, and not easily roused. Talks incoherently. Some muttering. Pulse 92. Respirations 20. Discontinue ammonia. Continue Quinia, and add to the treatment as follows:—

Tart. ant. et Pot. grs. iij,

Tinct. opii ʒss,

Aqua ℥ij,

ʒj every two hours, omitting if it occasion nausea.

April 8.—Was quite delirious during night, endeavoring to get out of bed, and, once, out of the window. This morning talks incoherently and mutters. Pulse 100, skin warm. Tongue moist and coated. Abdomen retracted. Abscess still discharges. No dejection. Respirations 20.

Expectorated this morning several solid sputa streaked with blood.

Treatment:—Sulph. Quiniæ, grs. ij, S. Morphiæ, gr. 1-6, every four hours. Carb. Ammoniæ, grs. 10, every two hours. Brandy, ℥ss, every two hours. Essence of beef and milk porridge, for diet.

P. M.—Continues to talk incoherently, and mutter. Skin cool and moist. Pulse 100. Abdomen retracted. One dejection, consistent, and of yellowish color. He has presented carphologia.

April 9. Sleeping. Pulse 88. Respirations 16. Slept tranquilly from midnight to 4, A. M. Skin moist at 4. Was actively delirious during the early part of night. Subsultus occasionally, while sleeping. No carphologia this morning. Copious discharge of urine. Abscess discharges but little, and swelling much diminished.

Continue treatment.

P. M.—Sleeping rather heavily. Pulse 88. Skin warm; easily roused. Says he is "first rate." Has seemed rational to-day. No carphologia. Urine deposited copious lateritious sediment.

April 10.—Comfortable night. Pulse 80, soft. Respirations 16. Appears perfectly rational. A few small furunculi on lower extremities. No dejection. Gums are spongy, and bleed readily on pressure.

Continue treatment, with omission of morphia.

April 11.—A copious sanguinolent discharge from the meatus auditorius took place last night, and now continues. Pus continues to be discharged from the opening below the ear. He is perfectly rational. Pulse 68. No disposition to somnolency.

Quinia, grs. ij, S. acid aromat. gtt. x, every two hours Carb. ammoniæ, grs. iv, Do. Port wine, ℥j, every four hours.

April 12.—Symptoms continue same as yesterday. Sanguinolent discharge continues. No dejection for several days. Abdomen retracted. Says he would relish a rare boiled egg. Let him have it, and give nutriment every two hours, porridge, or chicken broth, or egg and toast. Continue wine, ℥j, every two hours, and discontinue medicine. Yeast poultice to ear.

April 13.—Comfortable. Abscess discharges pus, and not blood. Portions of sloughing cellular tissue protrude from the opening. No dejection. Continue treatment, and give enema of warm water this afternoon, if no dejection.

April 14.—Improving. Two enemas administered yesterday have been retained. No dejection for several days.

Repeat enema every four hours, until dejection occurs, other treatment consisting wholly of wine.

April 15.—Improving. Slough of cellular tissue as large as the end of a finger protrudes from the orifice below the ear. No dejections, notwithstanding repeated injections. Abdomen soft and undistended. Abscess discharges freely, both from meatus, and below the ear.

P. Rhei, grs. xv,

P. Aromat, grs. ij, every four hours, until dejection.

April 16.—Had two dejections yesterday after second dose of Rheubarb, large, thin and yellow.

April 19.—Continues to convalesce; swelling under ear quite gone. Small discharge continues.

April 20.—Continues convalescent. Formation of matter has taken place on calf of leg, and is nearly ready to be discharged.

April 25.—Continues convalescent. From this date until May 10th, he was attended by Dr. J. S. Trowbridge, during my absence from the city. The discharge continued from the neck, through a fistulous opening, which was laid open by Prof. Hamilton, on the 28th of April.

On the 18th of May the following record was made: This patient has continued to convalesce, and is progressively improving. Discharge of pus from below the ear continued until a few days since. Purulent discharge from meatus still continues. He walks out daily, appetite is good, and, with exception of the purulent discharge just mentioned, (which is small and daily diminishing,) has no ailment.

Left Hospital a few days after the date of the foregoing record.

*Remarks:* Clinical reports, embracing the record of daily symptoms, are apt to be considered, by the reader, tedious, but it is obvious that, frequently, in this way alone can full justice be done, by the reporter, to the history and treatment of individual cases. In the foregoing report, we have practiced some abbreviation, retaining all that seemed essential for the present purpose. The case has been selected from a considerable number of fever cases, occurring at the Hospital, as affording an instance of an important complication, and, at the same time, furnishing an illustration of the series of phenomena presented in Typhus, together with the principles of treatment pursued by the reporter.

As a complication of continued fever, Parotis is, so far as the writer's observation goes, infrequent, but the fact of its occasional occurrence is well known to observers of much experience. It is a complication modifying somewhat the character and progress of the febrile disease, and adding considerably to the probabilities of an unfavorable termination. The propriety of designating the case one of *Typhus,* is not only apparent from its gravity, and the early development of *typho-mania,* but is sustained by the presence of criteria which are considered distinctive by those who consider *Typhus* and *Typhoid* fevers as essentially distinct. These criteria (present in this case,) are the characters of the eruption, to wit, *not elevated, small, the redness not disappearing on pressure;* and the absence of diarrhœa, abdominal tenderness, and marked meteorism. The absence of the latter symptoms would indicate that the intestinal lesions so frequent in continued fever, (and in the opinion of some exclusively pertaining to the so-distinguished typhoid fever,) were not prominent, or were altogether absent in this instance. With reference to the treatment, it will be perceived that the chief object was to sustain the vital forces, and, thus, to carry the patient safely through the disease. This, in the opinion of the writer, is the end which the practitioner should propose to himself in cases of fever not presenting complications requiring special treatment, such as visceral inflammation, apoplectic coma, etc. In the present condition of our Science it is in vain to undertake to *cure continued fever,* i. e. to arrest or limit its career, but we must be content with what is calculated to promote its favorable issue. In securing this result, the practitioner is by no means to be inactive. Positive measures are to be employed, and these consist chiefly in the judicious administration of tonics, stimulants and nutritious diet. Other collateral measures are also to be employed, which are to be adapted to particular circumstances belonging to the history of each case. Of the latter character, in this case, were those called for by the active delirium of the patient. To control this troublesome, and sometimes grave feature, we have found antimony, in combination with opium or morphia, to be most efficient. We have employed this combination more of late than heretofore, and in other cases more freely than in the one under present consideration; and we can add our testimony to that of Dr. Graves, of Dublin, of its remarkable power in the delirium of fevers. The reader will observe that in one or two occasions only during the progress of this case were cathartics or even laxatives prescribed. In our practice, of late years, we have abandoned, in a great degree, the use of this class of remedies in fever, and we think we have reason to feel satisfied with such a course. We have not found any detriment in permitting the bowels to go unmoved from one to three days ordinarily, and when any aid is necessary, simple enemas appear to us usually to be sufficient. We have observed perfectly natural evacuations during the course of fever, in which the intestinal mucus membrane has been left undisturbed by medicinal irritants. There is an extreme in either direction, which the judicious practitioner will seek to avoid, but if compelled to choose between the evils of constipation, and over-catharsis, in fever, we should be disposed to elect the former.

It will be observed that mercurials were not employed. We are unable to perceive any rational or analogical indications for their use in fever, except it be complicated with internal inflammation; and the experience of several years has confirmed the propriety of withholding them, except called for by circumstances other than the existence of fever.

Case III.—*Pericarditis with Effusion. Physical Diagnosis. Autopsical Appearances.*

The interest and value of this case relate chiefly to the combination of rational and physical signs which it presents, and the diagnosis based thereupon, taken in connection with the appearances found after death. Details not relevant to these points, will, accordingly be omitted.

Martin McGowan, aged 49, Irish, married, cooper, entered February 1st, 1849.

*History:* States that he has been ill eight weeks. Had good health previously. Was attacked with lancinating pains in left chest, accompanying each inspiration. Pains continued for two weeks. Has had no pain since, but shortness of breath. Had cough at first, which was extremely painful. Cough continues. Expectoration is slight. Has not been confined to the bed. Has had no medical treatment.

*Present Symptoms:* Respiration short and hurried. Unable to sleep, from embarrassed respiration. Walks about room with difficulty, owing to shortness of breath. Œdema of face and lower extremities. Urine as abundant as in health; appetite tolerable. Tongue coated and moist. Pulse 120, soft and feeble; skin cool and moist. What sleep he obtains is refreshing, and without bad dreams. His habits have been intemperate.

*Physical Examination:*—Good resonance on percussion over the upper third of each side, slight relative dullness over upper third of left side. Flatness in middle third of left side, over a triangular space, bounded on the left by nipple, on the right by the left margin of sternum; apex of triangle about three inches above the line of the nipple; base, at bottom of chest. Sibilant and sonorous rales over both sides. Posteriorly, in left interscapular region, loud, sibilant respiration. Sibilant respiration in right interscapular region, but less loud than in left. Resonance in both interscapular regions uniform and good. No impulse of heart discernible. Sounds of heart very feeble, and without murmur.

In view of the foregoing physical signs, taken in connection with the symptoms and history, the diagnosis is *Pericarditis with effusion.*

Treatment: Copaiva mixture; solution of nitras Potassæ, and blister to pracordial region.

The dyspnœa continued, at times considerable, and at times relieved. Frequently he was obliged to set up much of the night from difficulty of breathing while lying down. The œdema continued. The decumbency was generally on the right side. Pulse most of the time 120, occasionally somewhat less accelerated.

On the 7th of March it is noted that evident enlargement of chest in præcordial region existed; impulse of heart inappreciable, and no heaving of the chest; first sound of heart dull and indistinct, second sound sharp and clear.

The treatment consisted of various diuretics—squill, digitalis, nitrate and sup. larirate of Potash; Hoffman's anodyne; stimulants in moderate quantities; and, occasionally, cathartics and vesication.

Death occurred, suddenly, on the 10th of March, while at stool.

*Autopsy twenty hours after death:*—

*Chest.* Pericardium contained, by estimation, a pint of fluid, transparent at upper part, and turbid at lower. Pericardium lining the pericardial sac opaque. Two patches of lymphatic exudation on the heart, one of the size of a dollar, the other of the size of a shilling piece. Heart moderately hypertrophied. Large coagulum in left anricle; small coagula in other cavities. Some old adhesions of pleura in left side; universal old adhesions in right chest. The left lung only was minutely examined. It was emphysematous, without tubercles, a portion overlapping the heart.

Other organs not examined.

Case IV. *Pleuritis with Effusion, Recovery; Aneurism of Aorta, Rupture into Pericardial Sac*; *Autopsy.*

John Brown, Irishman, aged 41, married, laborer, entered February 21st, 1849.

*History:* States that he was well up to about eight weeks since. Never had any illness previously. He was attacked with cough, and acute lancinating pain in right chest, chills, loss of appetite, thirst, and heat of skin. Was confined to the bed, for the most part, for three weeks. Then he attempted to go to work, and labored eight days. Had no medical treatment during the three weeks he was confined to bed, and has had none since, up to time of entering Hospital. After leaving off work, which he was compelled to do, he has kept in house, and much of the time in bed. Has had cough and expectoration; pains in chest pretty constant, beneath sternum, also, coming and going, in other parts of chest; pains in left shoulder and left hip. No chills of late. Appetite poor. Is put out of breath by exercise. No hæmoptysis. Sputa have never been bloody, nor of a red color. Pulse 100. Temperature of skin somewhat raised.

*Physical Examination:* Marked dullness on percussion over the right chest, anteriorly, laterally, and posteriorly. Respiration in the infra clavicular region of right side, tubular. Expiration prolonged, and more pronounced than the inspiration. Absence of respiratory murmur in middle and inferior thirds of right chest anteriorly and laterally. Posteriorly, on right side, at inferior angle of scapula, sonorous rale. On left side, good resonance on percussion; respiration somewhat exaggerated.

He states that when first attacked, pain in right side, and cough, came on simultaneously, and that he had difficulty of respiration at the same time. Pain in right chest still continues, but is much less severe than heretofore. Tongue is coated and yellow. One dejection yesterday. Urine scanty.

Treatment:—Sup. Tart. Potash ℥ss.

Aquæ Ojss.

To be drank during the day,

Pulv. Digitalis, gr. j.
" Scillæ, grs. ij.
Massa Hydrarg. gr. j.
Every six hours.

23d.—Reports more comfortable. Has expectorated since last night about six ounces of muco-purulent sputa, with considerable serous fluid. Urine increased.

Treatment continued.

25th.—Resonance on percussion on the right side, above the nipple, is pretty good. Some relative dullness. Laterally, on same side, good for two or three inches below the line of the nipple. Posteriorly, good throughout. No respiratory murmur, but, occasionally, a slight sonorous rale in the infra clavicular region of right chest. Sounds of heart loudly audible over right chest. Respiratory murmur on left side intense. Posteriorly, on right side, respiration tubular. Tenderness on percussion of inferior portion of right chest.

Treatment: Blister over lower part of right chest.

28th.—Resonance over right chest improving; it is now good two inches below the line of the nipple.

March 3d.—Reports better. Cough and expectoration much diminished. Feeble respiratory murmur, and sonorous rale, at inferior part of chest, anteriorly and posteriorly. Chest on right side appears somewhat contracted, at inferior portion, both anteriorly and posteriorly.

From this date to March 14th, the record, daily, is, improving. Some cough continued, and occasionally occurring in pretty violent paroxysms. On the 14th, it is noted that evident contraction of the right side existed, the shoulders being depressed, and scapula nearer the spine than on the other side.

21st.—Reports better. Cough diminished, and expectorates but little.

26th.—Reports, as before, quite well. The treatment for several days had been S. Quiniæ, gr. j, S. Ferri, grs. jj, three times daily. He has for some time been in the habit of daily walking in the open air.

He appeared quite as well as usual during this day, walked in the yard, and was seen, at 5, P. M., to come out of a grocery opposite the Hospital. He ate his supper as usual, was sitting by the fire talking with other patients, directly after leaving the supper table, when, in the midst of conversation, he suddenly started, and fell dead upon the floor.

*Autopsical appearances eighteen hours after death:* Body presented largely developed chest and limbs, with considerable embonpoint. Head was first opened. On section of the scalp, fluid blood flowed freely and copiously. After removing the calvarium, blood escaped freely from the orifices of the vessels ruptured by separation of the skull. Vessels of dura mater greatly engorged. Some serum escaped on dividing the dura mater. The arachnoid membrane was distended, and rendered opaque by sub-arachnoid serous effusion, especially marked between the convolutions. Serum flowed pretty freely on section of arachnoid. Superficial veins of brain much congested. On section of cerebral substance, it presented but few red points. The substance of brain appeared ex-sanguine. Lateral ventricles contained, each, about half an ounce of serum, slightly opaque. In chest, adhesions over several circumscribed portions in each side. Pericardium largely distended, and filled with coagula and serum. Large aneurism of aorta, which had ruptured into pericardial cavity.

*Remarks:* The account of the condition of the pleural cavities in the ecord of autopsical appearances is meagre, the aneurism, when discovered, having engrossed the attention. The recovery from the pleuritis was, however, complete, the effused fluid having been entirely absorbed. The appearances within the cranium are worthy of note, showing, as they did, [illegible]ate of vascular engorgement and effusion, which appeared sufficient to account for death. Indeed, before the chest was opened, the expectation was, that the conclusion from the autopsy would be that the patient died of simple apoplexy. And, in fact, this may be a correct view of the case. The immediate compression of the heart by the effusion into the pericardium, may be regarded as having induced death by congestion of brain, prior to the induction of fatal consequences by the cessation of the circulation in general. Death occurred more instantaneously than if from mere cessation of the heart's action. The action of the heart may, for a time, be almost intermitted, as in syncope, without a fatal result. But in this instance there occurred a sudden and complete compression of the cavities of the heart, inducing, at once, non-circulation, and congestion especially of the brain—hence, the instantaneous death. It will be observed that although there is no reason to suppose there existed any cerebral affection prior to the moment of the decease of the patient, there existed not only engorgement, but considerable effusion, both into the lateral ventricles and the sub-arachnoid cellular tissue. Hence, these appearances do not of themselves denote inflammation. They may, as was probably the case in this instance, occur after death, i. e. the effusion.

The existence of aneurism was not suspected before death. Occupying a situation beneath the sternum, the aneurismal tumor did not occasion any physical signs attracting notice during the examinations of the chest. It is somewhat surprising that, in listening to the sounds of the heart, the *bruit* of the tumour, if it existed, should have eluded notice. The examinations, however, after the diagnosis of pleuritis was made, were particularly directed to the middle and inferior thirds of the chest.

It is a matter of surprise that a disease of this kind, which must have existed for a considerable period, should have occasioned little or no perceptible difficulty. Up to his attack of pleuritis, eight weeks before his entrance, the patient stated that his health was perfect, and that he was able to do manual labor without inconvenience.

Case VI.—*Chronic Pleuritis with Effusion. Recovery.* Daniel Mahony, aged 23, Irishman, laborer, unmarried. Entered March 26th, 1849.

*History:* He was taken ill about the first of January last. Enjoyed good health previously. Was attacked with acute, lancinating pain in inferior portion of right chest, most severe in inspiration. Had cough, which occasioned extreme pain. Kept about, but did not labor, for twenty days, before applying for medical aid. He then applied to a German irregular practitioner, who bled him and prescribed a blister. Has had no pain since blister was applied. Has been able to be about most of the time since, but not to do any work.

*Present Symptoms:*—Slight cough and expectoration. Appetite poor. Bowels costive. Complains of debility. Exercise occasions panting. No pain. Urinates freely. Pulse 108, rather small. Says he has a slight chill almost every afternoon, followed by heat, without perspiration. Complains of thirst. Tongue furred.

*Physical Examination:*—Greater expansion of left chest, on inspiration, than of right. Marked enlargement of right side apparent on posterior view. Flatness on percussion over lower and middle third of right chest, anteriorly, and posteriorly. Absence of respiratory murmur in portion of right chest presenting flatness on percussion. No œdema of affected side, nor enlargement of sub-cutaneous veins. Sounds of heart, and impulse, normal. Respiration of upper third of right chest feeble and tubular; in left chest supplementary. Aspect of patient pallid.

Treatment:—Pr. t. Chlor. Hydrarg, gr. j,
Pulv. Digitalis, gr. j,
" Scillæ, grs. jj, every four hours. Sup. Tart. Potassæ, ℥ss, to be taken in solution during twenty-four hours. Gruel, or porridge, for diet.

29th.—No material alteration of symptoms. Urine deposits lithatic sediment. Cont. Treatment, and add blister to chest, 6x4.

31st.—Reports about the same. Had twelve dejections yesterday, and one this morning, copious and watery. Thinks quantity of urine unaffected. Pulse 76. Resonance over right chest, anteriorly, good, as low as the nipple. Cont. treatment, adding brandy ℥ss, every four hours. He has been a moderate drinker.

April 1.—Distinct evidences of ptyalism. Cont. treatment, omitting calomel.

4th.—Has continued to have two or three dejections daily, large and watery. Urine not increased. The visible enlargement of chest has entirely gone. Resonance as low as nipple. Pulse 100. Cont. treatment, and apply blister, 4x4, posteriorly.

7th.—Reports feeling stronger. Watery dejections continue. Sits up half of the day. Pulse 68. Results of physical examination same as on the 1st inst. Cont. treatment, adding a little jalap to the Sup. tart. Potassæ.

9th.—Jalap omitted, and tonic solution, S. Ferri and S. Quiniæ, prescribed,

13th.—Watery dejections continue. Complains of pain, and of coldness in abdomen. Discontinue the Sup. Tart. Pot., and give S. Morphiæ, gr. 1-6, every four hours. Aspect continues pallid. Dullness below nipple continues. Intercostal spaces depressed. Liver extends into left hypochondrium.

Treatment: Lugol's Solution of Iodine, gtt. x, three times daily. Continue Iron and Quinia solution.. Apply blister to inferior part of chest, 4x4.

20th.—Has reported, daily, better. Dejections still more frequent and watery than in health. Cough and expectoration have entirely disappeared. Flatness below nipple continues. Cont. Treatment.

From this date, this patient progressively improved, and on the 14th of May the following record of physical signs was made:—Right chest presents marked contraction. The shoulder is depressed. Anteriorly, the chest is flattened, leaving the clavicle more prominent than on the other side. Marked depression in supra-spinous region, the interscapular spaces remaining the same on both sides. There is now resonance on percussion an inch below the nipple on the right side. Dullness extends, laterally, somewhat higher. The pitch of resonance over the right side, generally, is higher than on the left side. He has no cough nor expectoration. No febrile movement. Occasionally he experiences a sharp pain in the right chest on deep inspiration. Bowels move twice daily, and still are somewhat liquid. Cont. medicine, (i. e. Iodine and tonic solution,) and apply vol. liniment to right chest morning and evening.

In a few days after this date he felt sufficiently well to leave the Hospital, and several weeks afterward he presented himself, as a visitor, and reported quite well.

*Remarks:* The history of this case presents no peculiarity worthy of especial note, except the direction of the diuretic remedies to the bowels instead of the kidneys, producing results as satisfactory as if they had operated on the latter organs. The case is reported as furnishing an illustration of the symptoms and physical signs before, and after recovery, and of the efficacy of the therapeutic measures employed.

